# Effect of religion on hypertension in adult Buddhists and residents in China: A cross-sectional study

**DOI:** 10.1038/s41598-018-26638-4

**Published:** 2018-05-29

**Authors:** Qingtao Meng, Ying Xu, Rufeng Shi, Xin Zhang, Si Wang, Kai Liu, Xiaoping Chen

**Affiliations:** Department of Cardiology, West China Hospital, Sichuan University, Chengdu, 610041 China

## Abstract

Correlation between religion and hypertension is worth investigating since they both influence many people. Compared to studies which quantify religion with indicators representing only restricted dimensions of religion, researches assessing religion as an integral is preferable while lacking. Moreover, religious behaviors have great potential to be generalized if they are proved to be mediator through which religion exerts effect. However, relevant evidence is limited. Therefore, this cross-sectional study recruited 1384 adult Tibetan Buddhists from two Buddhist institutes in the Sichuan Province of China, and enrolled 798 adult Tibetan residents from nearby villages/towns. Each participant received a questionnaire, physical examination, and blood biochemistry tests. Buddhist effect on hypertension was investigated. The effects of uniquely Buddhist behaviors on hypertension were analyzed. The hypertensive risk of the Tibetan Buddhists is significantly decreased by 38% than Tibetan residents. As a Buddhist behavior, vegetarian diet highly approximates to be protective for Tibetan hypertension. As another Buddhist behavior, longer Buddhist activity participation time is associated with decreased prevalence of hypertension as well as lower blood pressure (BP) by analyzing subgroup of 570 Buddhists. Therefore, the protective role of religion on hypertension is suggested, and the religious behaviors are mediators which may be applied to general population.

## Introduction

Religion is defined as a set of beliefs concerning the cause, nature and purpose of the universe; it usually involves devotional and ritual observances and often contains a moral code for the conduct of human affairs^1^. It is designed to facilitate closeness to the transcendent and to foster an understanding of one’s relationship with and responsibility to others^2^. Sizeable majorities claim to be religious worldwide, and the global average proportion of religious people is 59% according to a recent survey^3^. As important aspects of human life, religion and medicine have been related in one way or another in all population groups since the beginning of recorded history^2^. Since there is evidence suggesting that religion may exert effects on believers both physically and psychologically, increasing attention has been paid to religion and health^4,5^.

As the most significantly modifiable risk factor for cardiovascular diseases, essential hypertension accounts for 208.1 million disability-adjusted life years (DALYs) and 10.4 million deaths worldwide^6^. As early as in the 1960s, research about religion and hypertension began to be reported^7^; subsequently, increasing numbers of studies were published during the following years. Most of them quantified religion by indicators such as the frequency of attendance at religious ceremonies^8–10^ and the extent of religiosity^11–13^. Since each indicator reflects only one dimension of religion, its representativeness of religion is unique and restricted. As a result, different indicators display inconsistent correlations with hypertension even when they are simultaneously surveyed in the same population^14^. Therefore, studies evaluating religion as an integrated whole are better able to determine the relationship between religion and hypertension since they can assess religion comprehensively, especially those comparing the hypertensive risk between full-time religious staff and ordinary residents. However, these studies are far from sufficient. In addition, if full-time religious staff members are found to have a lower risk of hypertension, whether religious behaviours are the mediators through which religion exerts its effects is worthy of investigation because those behaviours have great potential to be applied to the general population. Additionally, the relevant evidence is limited^15^; thus, more studies are required.

Therefore, by comparing the hypertensive risk of Tibetan Buddhists with that of the general population living in Sichuan Province in China, this research aimed to test the hypothesis that religion plays a role in hypertension. Further, once it was established that a difference in the hypertensive risk between the two populations existed, whether religion-related behaviours were mediators of that difference was investigated.

## Results

### Comparison of demographic data and hypertensive characteristics between Buddhists and residents

#### Demographic data and hypertensive characteristics of the two populations

**Figure 1:**
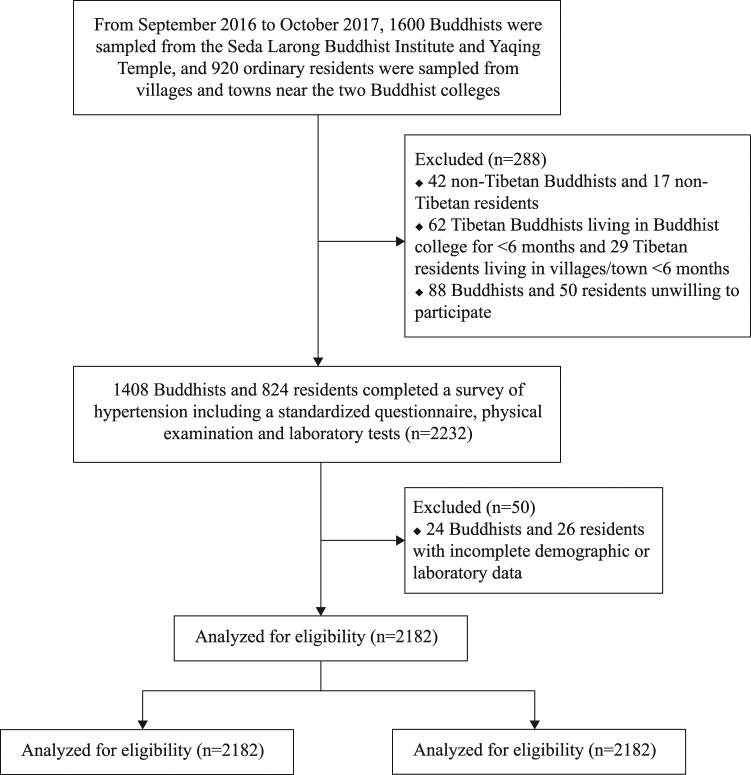
Flow chart of the study. The process of participant enrolment, inclusion and exclusion is displayed in this flow chart.

A total of 1600 Buddhists and 920 residents of nearby towns and villages were initially recruited. As the flowchart demonstrates (Fig. 1), after excluding non-Tibetans (n = 59), those living in the area for <6 months (n = 91), and those unwilling to participate in study (n = 138), 1408 Buddhists and 824 residents completed the survey on hypertension. After further exclusion of those with incomplete demographic or laboratory data (n = 50), there were 1384 Buddhists and 798 residents ultimately included for analysis.

As shown in Table 1, systolic BP (126.4 ± 24.2 mmHg vs 138.9 ± 25.7 mmHg, P < 0.01), diastolic BP (76.6 ± 14.7 mmHg vs 84.1 ± 15.5 mmHg, P < 0.01), triglycerides (1.12 ± 0.58 mmol/L vs 1.32 ± 0.77 mmol/L, P < 0.01), low density lipoprotein cholesterol (LDL-C) (2.53 ± 0.88 mmol/L vs 2.76 ± 0.79 mmol/L, P < 0.01), blood glucose (5.24 ± 1.21 mmol/L vs 5.63 ± 1.88 mmol/L, P < 0.01) and the prevalence of diabetes mellitus (1.7% vs 8.7%, P < 0.01) were significantly lower in Tibetan Buddhist monks and nuns than in ordinary Tibetan residents. The Buddhist monks and nuns do not smoke or consume alcohol and engage in almost no exercise.

**Table 1:** Demographic characteristics of Tibetan Buddhists and ordinary Tibetan residents.

	Tibetan Buddhist monks and nuns			Ordinary Tibetan residents			P value^#^
	Total	Male	Female	Total	Male	Female	
N	1384	664	720	798	367	431	0.374
Age (years)	45.1 ± 15.6	43.4 ± 15.7	46.7 ± 15.3*	45.8 ± 15.4	45.5 ± 16.7	46.0 ± 14.2	0.316
Family history of hypertension (%)	4.9	5.4	4.4	8.5	13.6	4.2*	0.001
Smoking (%)	0.0	0.0	0.0	1.5	3.3	0.0*	0.000
Alcohol consumption (%)	0.0	0.0	0.0	0.5	1.1	0.0*	0.018
Physically active (%)	0.0	0.0	0.0	0.9	1.4	0.5	0.001
BMI (kg/m^2^)	24.7 ± 4.2	24.8 ± 4.2	24.5 ± 4.2	24.4 ± 4.0	24.6 ± 4.0	24.2 ± 4.0	0.086
Overweight or obese (%)	51.4	52.6	50.3	49.3	53.7	45.6*	0.364
SBP (mmHg)	126.4 ± 24.2	128.9 ± 24.2	124.0 ± 24.1*	138.9 ± 25.7	142.4 ± 24.6	135.9 ± 26.2*	0.000
DBP (mmHg)	76.6 ± 14.7	78.9 ± 15.4	74.6 ± 13.7*	84.1 ± 15.5	87.3 ± 15.6	81.3 ± 14.8*	0.000
Pulse (beats/min)	83.4 ± 14.1	83.92 ± 14.97	83.13 ± 13.47	84.3 ± 12.4	84.34 ± 12.69	84.34 ± 12.30	0.169
TG (mmol/L)	1.12 ± 0.58	1.14 ± 0.61	1.10 ± 0.53	1.32 ± 0.77	1.34 ± 0.74	1.30 ± 0.78	0.000
TC (mmol/L)	4.40 ± 1.11	4.19 ± 1.04	4.65 ± 1.15*	4.55 ± 1.01	4.43 ± 1.08	4.63 ± 0.96	0.064
LDL-C (mmol/L)	2.53 ± 0.88	2.40 ± 0.82	2.69 ± 0.93*	2.76 ± 0.79	2.75 ± 0.80	2.77 ± 0.79	0.001
HDL-C (mmol/L)	1.27 ± 0.31	1.17 ± 0.27	1.38 ± 0.32*	1.34 ± 0.34	1.15 ± 0.26	1.45 ± 0.34*	0.006
Blood glucose (mmol/L)	5.24 ± 1.21	4.94 ± 1.20	5.51 ± 1.15*	5.63 ± 1.88	5.37 ± 2.16	5.85 ± 1.56*	0.000
Diabetes mellitus (%)	1.7	1.7	1.6	8.7	10.7	7.6	0.000

*P < 0.05 when male vs female; ^#^P < 0.05 when Tibetan Buddhists vs ordinary Tibetan residents; BMI: Body mass index; SBP: Systolic blood pressure; DBP: Diastolic blood pressure; TG: Triglycerides; TC: Total cholesterol; LDL-C: Low density lipoprotein cholesterol; HDL-C: High density lipoprotein cholesterol; Overweight: 24 ≤ BMI < 28 kg/m^2^; Obese: BMI ≥ 28 kg/m^2^.

#### Awareness, treatment and control of hypertension in the two populations

Table 2 demonstrates the lower hypertensive prevalence in Tibetan Buddhists than in ordinary Tibetan residents (23.3% vs 41%, P < 0.01), and the difference still existed after age standardization (20.0% vs 33.2%, P < 0.01). In both populations, there was a significantly higher hypertensive prevalence in males than in females. Similarly, monks/nuns had significantly lower rates than residents in terms of both hypertension awareness (43.5% vs 52.6%, P < 0.05) and treatment (30.1% vs 41.3%, P < 0.01). However, the blood pressure control rate in treated hypertensive patients was significantly higher in Tibetan monks/nuns than in ordinary Tibetan residents (20.6% vs 8.9%, P < 0.05).

**Table 2:** Awareness, treatment and control of hypertension in the two populations.

	Tibetan Buddhist monks and nuns			Ordinary Tibetan residents			P value^#^
	Total	Male	Female	Total	Male	Female	
Hypertensive patients (n) and prevalence of hypertension (%)	322 (23.3)	174 (26.2)	148 (20.6)*	327 (41.0)	173 (47.1)	154 (35.7)*	0.000
Age-standardized hypertension prevalence ^‡^(%)	20.0	23.9	16.4*	33.2	41.4	25.5*	0.000
Hypertension awareness rate (%)	43.5	48.9	37.2*	52.6	56.6	48.1	0.023
Hypertension treatment number (n) and rate (%)	97 (30.1)	58 (33.3)	39 (26.4)	135 (41.3)	81 (46.8)	54 (35.1)*	0.003
Hypertension control rate in all individuals (%)	6.2	8.6	3.4	3.7	4.1	3.2	0.151
Hypertension control rate in treated patients (%)	20.6	25.9	12.8	8.9	8.6	9.3	0.012

*P < 0.05 when male vs female; ^#^P < 0.05 when Tibetan Buddhists vs ordinary Tibetan residents; BMI: Body mass index; SBP: Systolic blood pressure; DBP: Diastolic blood pressure; TG: Triglycerides; TC: Total cholesterol; LDL-C: Low density lipoprotein cholesterol; HDL-C: High density lipoprotein cholesterol; Overweight: 24 ≤ BMI < 28 kg/m^2^; Obese: BMI ≥ 28 kg/m^2^.

#### Analysis of the risk factors for hypertension

As shown in Table 3, in univariate logistic regression analysis, older age (OR 1.065, CI 1.057–1.072, P < 0.01), male sex (OR 1.426, CI 1.186–1.715, P < 0.01), a family history of hypertension (OR 2.292, CI 1.615–3.252, P < 0.01), higher body mass index (BMI) (OR 1.101, CI 1.075–1.127, P < 0.01), diabetes mellitus (OR 2.177, CI 1.132–4.187, P < 0.05), and higher total cholesterol level (OR 1.699, CI 1.456–1.983, P < 0.01) were all independent risk factors for hypertension, while being a Buddhist monk/nun was a significant protective factor against hypertension (OR 0.437, CI 0.362–0.527, P < 0.01).

**Table 3:** Logistic regression analysis of hypertension.

	Univariate model			Multivariate model 1			Multivariate model 2		
	OR	95% CI	P value	OR	95% CI	P value	OR	95% CI	P value
Age (1 year)	1.065	1.057–1.072	0.000	1.072	1.064–1.081	0.000	1.074	1.057–1.091	0.000
Gender (male vs female)	1.426	1.186–1.715	0.000	1.843	1.469–2.312	0.000	1.566	0.996–2.462	0.052
BMI (1 kg/m^2^)	1.101	1.075–1.127	0.000	1.098	1.069–1.128	0.000	1.133	1.080–1.190	0.000
Family history of hypertension (yes vs no)	2.292	1.615–3.252	0.000	2.926	1.915–4.472	0.000	5.054	2.246–11.373	0.000
Physically active (yes vs no)	1.775	0.396–7.954	0.453	0.543	0.058–5.103	0.593	4.755	0.369–61.264	0.232
Smoking (yes vs no)	1.182	0.355–3.940	0.785	0.355	0.086–1.456	0.150	1.400	0.273–7.169	0.687
Alcohol consumption (yes vs no)	2.366	0.333–16.835	0.390	1.935	0.190–19.733	0.577	4.719	0.419–53.168	0.209
Diabetes mellitus (yes vs no)	2.177	1.132–4.187	0.020	—	—	—	0.623	0.212–1.832	0.390
TC (1 mmol/L)	1.699	1.456–1.983	0.000	—	—	—	1.161	0.932–1.447	0.182
Buddhist monks and nuns (vs ordinary residents)	0.437	0.362–0.527	0.000	0.348	0.276–0.438	0.000	0.620	0.388–0.991	0.046

Multivariate regression equation model 1 includes confounding factors such as age, gender, BMI, family history of hypertension, smoking, alcohol consumption and physical activity.

Multivariate regression equation model 2 includes confounding factors such as age, gender, BMI, family history of hypertension, smoking, alcohol consumption, physical activity, TC, and diabetes mellitus.

In model 1 (Table 3), after controlling for some confounding factors including age, gender, BMI, family history of hypertension, physical activity, smoking status, and alcohol consumption habits, the risk of hypertension was still significantly lower in Buddhist monks/nuns than in ordinary residents (OR 0.348, CI 0.276–0.438, P < 0.01).

In model 2 (Table 3), when diabetes mellitus and total cholesterol were controlled in addition to the above confounding factors, Buddhist monks/nuns still had a 38% lower risk of hypertension than ordinary residents (OR 0.620, CI 0.388–0.991, P < 0.05).

#### Analysis of blood pressure control under antihypertensive treatment

Among those on treatment (n = 232), there were respectively 97 and 135 Buddhists and ordinary residents. As in Table 2, 20 of the treated Buddhists and 12 of the residents under treatment had BP controlled (20.6% vs 8.9%, P < 0.05). In addition, in Logistic regression analysis, treated Buddhists were more likely to have BP controlled when compared with residents, no matter in univariate analysis (OR = 2.662, 95% CI 1.232–5.752, P < 0.05) or multivariate analysis (OR = 2.802, 95% CI 1.203–6.528, P < 0.05) with age, gender, BMI, and family history of hypertension adjusted. Since the total sample size of subjects under antihypertensive treatment was just 232, just the basically confounding factors were controlled.

### Influence of Buddhist behaviours on hypertension

According to the investigation into the behaviours of both Buddhists and residents, vegetarian diet and Buddhist activity are unique to the Tibetan Buddhists. Therefore, the effects of those behaviours on hypertension in the two populations were analysed.

#### Influence of vegetarian diet on hypertension

Vegetarians in this study were lacto-ovo vegetarians who ate meat and/or fish less than once per month and who consumed dairy products more than once per month^16^. The proportion of vegetarian in Tibetan Buddhists is as high as 68.1%, while that in ordinary residents is just 22.7%. Furthermore, in the non-vegetarian monks/nuns, the prevalence of eating meat at frequencies of <3 times/week, 3–5 times/week and ≥5 times/week are 64.7%, 14.5% and 20.8%, which are significantly different from the prevalences in ordinary residents (16.1%, 44.4%, and 39.5%, respectively).

As demonstrated in Table 4, in all subjects, vegetarians had significantly lower levels of several cardiovascular factors than non-vegetarians, such as systolic BP (SBP) (131.34 ± 25.62 mmHg vs 136.24 ± 25.67 mmHg, P < 0.01), diastolic blood pressure (DBP) (79.84 ± 15.25 mmHg vs 82.45 ± 15.69 mmHg, P < 0.01), TG (1.09 ± 0.57 mmol/L vs 1.26 ± 0.70 mmol/L, P < 0.01), LDL-C (2.48 ± 0.85 mmol/L vs 2.67 ± 0.81 mmol/L, P < 0.01), prevalence of hypertension (29.6% vs 38.1%, P < 0.01) and prevalence of diabetes mellitus (1.9% vs 7.1%, P < 0.01). When vegetarians were compared with those who ate meat ≥5 times/month, a difference in the prevalence of overweight/obesity appeared (48.1% vs 55.4%, P < 0.05).

**Table 4:** Clinical data of vegetarians and non-vegetarians.

	Vegetarians	Non-vegetarians		P value^*^	P value^‡^
		Total	Meat-eating ≥ 5 times/month		
N	875	937	266	—	—
Gender (male, %)	60.9	44.2	41.4	0.000	0.000
Monks/nuns (%)	79.5	34.8	16.2	0.000	0.000
Age (year)	43.56 ± 15.72	45.41 ± 15.14	45.36 ± 14.16	0.011	0.076
BMI (kg/m^2^)	24.32 ± 4.10	24.67 ± 4.22	24.91 ± 4.06	0.086	0.043
Overweight/obese (%)	48.1	51.1	55.4	0.229	0.046
SBP (mmHg)	131.34 ± 25.62	136.24 ± 25.67#	135.54 ± 24.37	0.000	0.018
DBP (mmHg)	79.84 ± 15.25	82.45 ± 15.69#	82.74 ± 15.04	0.000	0.006
Blood glucose (mmol/L)	5.16 ± 1.11	5.60 ± 1.84#	6.12 ± 2.40	0.000	0.000
TG (mmol/L)	1.09 ± 0.57	1.26 ± 0.70#	1.44 ± 0.78	0.000	0.002
TC (mmol/L)	4.34 ± 1.07	4.47 ± 1.03	4.74 ± 1.40	0.082	0.049
LDL-C (mmol/L)	2.48 ± 0.85	2.67 ± 0.81#	2.97 ± 1.09	0.001	0.002
HDL-C (mmol/L)	1.27 ± 0.30	1.32 ± 0.33	1.31 ± 0.34	0.053	0.400
Hypertension prevalence (%)	29.6	38.1	38.3	0.000	0.008
DM prevalence (%)	1.9	7.1	20.9	0.000	0.000

*P < 0.05 when vegetarians vs non-vegetarians; ^‡^P < 0.05 when vegetarians vs meat-eating ≥ 5 times/week; ^#^P < 0.05 in analysis of covariance, when vegetarians vs non-vegetarians.

BMI: Body mass index; SBP: Systolic blood pressure; DBP: Diastolic pressure; TG: Triglycerides; TC: Total cholesterol; LDL-C: Low density lipoprotein cholesterol; HDL-C: High density lipoprotein cholesterol.

Since vegetarians were younger than non-vegetarians, analysis of covariance was used to adjust for age. Nevertheless, vegetarians were still found to have lower SBP and DBP than non-vegetarians.

As shown in Table 5, when logistic regression analysis was adjusted for confounding factors that might interfere with the results, including age, gender, BMI, family history of hypertension, physical activity, smoking status, and alcohol consumption habits, vegetarian was a significantly protective factor against hypertension (OR 0.691, CI 0.542–0.881, P < 0.01). After also adjusting for diabetes mellitus and total cholesterol (TC), the ability of vegetarianism to decrease the hypertensive risk still nearly reached significance (OR 0.660, CI 0.424–1.026, P = 0.065).

**Table 5:** Logistic regression analysis of vegetarianism and hypertension.

	OR	95% CI	P value	Multivariate model 1			Multivariate model 2		
				OR	95% CI	P value	OR	95% CI	P value
Age (year)	1.065	1.057–1.072	0.000	1.083	1.073–1.093	0.000	1.080	1.062–1.098	0.000
Gender (male vs female)	1.426	1.186–1.715	0.000	1.402	1.098–1.790	0.007	1.394	0.873–2.226	0.164
BMI (kg/m^2^)	1.101	1.075–1.127	0.000	1.100	1.068–1.132	0.000	1.134	1.079–1.192	0.000
Family history of hypertension	2.292	1.615–3.252	0.000	2.670	1.741–4.096	0.000	4.956	2.156–11.391	0.000
Physically active	1.775	0.396–3.252	0.453	0.678	0.065–7.102	0.746	5.445	0.396–74.925	0.205
Smoking	1.182	0.355–3.940	0.785	0.506	0.121–2.117	0.351	1.692	0.334–8.584	0.526
Alcohol consumption	2.366	0.333–16.835	0.390	2.954	0.271–32.237	0.374	6.136	0.524–71.788	0.148
Diabetes mellitus	2.177	1.132–4.187	0.020	—	—	—	0.574	0.191–1.725	0.322
Total cholesterol	1.699	1.456–1.983	0.000	—	—	—	1.274	0.999–1.625	0.051
Vegetarian	0.683	0.561–0.831	0.000	0.691	0.542–0.881	0.003	0.660	0.424–1.026	0.065

Model 1 includes confounding factors such as age, gender, BMI, family history of hypertension, smoking, alcohol consumption, physical activity.

Model 2 includes confounding factors such as age, gender, BMI, family history of hypertension, smoking, alcohol consumption, physical activity, total cholesterol, and diabetes mellitus.

#### Influence of participation in Buddhist activity on hypertension

Buddhist activity is a routine practised by Tibetan Buddhists and not by ordinary residents.

The analysis in this section was restricted to 570 Buddhist monks/nuns who had complete data regarding their time spent engaging in Buddhist activity in the Seda Larong Buddhist Institute. In total, 144 of these subjects were hypertensive patients, and 426 of them had normal BP.

These participants were divided into 4 groups according to the Buddhist activity time quartiles (Q1: < 8 h/d; Q2: ≥ 8 h/d < 10 h/d; Q3: ≥ 10 h/d < 11 h/d; Q4: ≥ 11 h/d). As seen in Table 6, compared with the other three groups, the Q1 group were significantly older (47.28 ± 17.09 years vs 39.55 ± 14.09 years vs 39.26 ± 15.19 years vs 40.47 ± 13.93 years, P < 0.01) and had higher levels of SBP (138.30 ± 28.45 mmHg vs 122.74 ± 21.98 mmHg vs 123.18 ± 23.94 mmHg vs 121.08 ± 19.42 mmHg, P < 0.01), DBP (83.71 ± 17.90 mmHg vs 72.96 ± 12.97 mmHg vs 72.59 ± 12.00 mmHg vs 72.27 ± 11.75 mmHg, P < 0.01), hypertension prevalence (53.4% vs 28.1% vs 18.5% vs 11.8%, P < 0.01), TG (1.38 ± 0.73 mmol/L vs 1.10 ± 0.63 mmol/L vs 1.05 ± 0.55 mmol/L vs 1.02 ± 0.50 mmol/L, P < 0.01), and TC (4.73 ± 1.39 mmol/L vs 4.25 ± 1.21 mmol/L vs 4.16 ± 1.31 mmol/L vs 4.13 ± 1.36 mmol/L, P < 0.01).

**Table 6:** Comparison among Buddhist activity time quartiles.

	Sum	Q1	Q2	Q3	Q4	P value
N	570	103	146	151	170	—
Buddhist activity time (h/d)	9.18 ± 2.08	5.71 ± 1.15^†‡∮^	8.26 ± 0.44^‡∮^	10.00 ± 0.04^∮^	11.35 ± 0.49	0.000
Age (year)	41.15 ± 15.16	47.28 ± 17.09^†‡∮^	39.55 ± 14.09	39.26 ± 15.19	40.47 ± 13.93	0.000
Male (n, %)	295 (51.8%)	51 (49.5%)	69 (47.3%)	87 (57.6%)	88 (51.8%)	0.325
BMI (kg/m^2^ ± SD)	25.49 ± 4.47	25.66 ± 4.51	25.72 ± 4.27	25.57 ± 4.79	25.15 ± 4.33	0.683
Overweight/Obese (n, %)	341 (59.8%)	73 (70.9%)	89 (61.0%)	85 (56.3%)	94 (55.3%)	0.056
SBP (mmHg)	125.17 ± 23.59	138.30 ± 28.45^†‡∮^	122.74 ± 21.98	123.18 ± 23.94	121.08 ± 19.42	0.000
DBP (mmHg)	74.60 ± 14.06	83.71 ± 17.90^†‡∮^	72.96 ± 12.97	72.59 ± 12.00	72.27 ± 11.75	0.000
Pulse rate (beats/min)	82.97 ± 14.14	82.27 ± 14.47	83.40 ± 14.01	84.26 ± 14.89	81.89 ± 13.36	0.456
Hypertension prevalence (n, %)	144 (25.3%)	55 (53.4%)^†‡∮^	41 (28.1%)	28 (18.5%)	20 (11.8%)	0.000
FBG (mmol/L)	4.83 ± 0.98	5.07 ± 1.53^‡∮^	4.81 ± 0.89	4.79 ± 0.93	4.78 ± 0.77	0.184
DM prevalence (n, %)	12 (2.4%)	3 (3.5%)	4 (3.0%)	3 (2.2%)	2 (1.3%)	0.687
TC (mmol/L)	4.29 ± 1.33	4.73 ± 1.39^†‡∮^	4.25 ± 1.21	4.16 ± 1.31	4.13 ± 1.36	0.008
TG (mmol/L)	1.12 ± 0.61	1.38 ± 0.73^†‡∮^	1.10 ± 0.63	1.05 ± 0.55	1.02 ± 0.50	0.000
LDL-C (mmol/L)	2.44 ± 0.97	2.70 ± 1.07^‡∮^	2.43 ± 0.89	2.35 ± 0.96	2.36 ± 0.96	0.056
HDL-C (mmol/L)	1.26 ± 0.69	1.43 ± 1.25^∮^	1.24 ± 0.34	1.24 ± 0.58	1.18 ± 0.41	0.083

^†^P < 0.05 vs Q2; ^‡^P < 0.05 vs Q3; ^∮^P < 0.05 vs Q4.

As shown in Table 7, logistic regression analysis revealed a significantly protective role of greater time spent participating in Buddhist activity against hypertension both in univariate analysis (Q2 vs Q1, OR 0.341, CI 0.201–0.579, P < 0.01; Q3 vs Q1, OR 0.199, CI 0.113–0.349, P < 0.01; Q4 vs Q1, OR 0.116, CI 0.063–0.213, P < 0.01) and in multivariate analysis, which adjusted for several confounding factors (Q3 vs Q1, OR 0.287, CI 0.105–0.785, P < 0.05; Q4 vs Q1, OR 0.078, CI 0.023–0.268, P < 0.01). After the exclusion of 44 hypertensive patients under antihypertensive treatment, multiple linear regression analysis was conducted among the remaining 526 Buddhist monks/nuns. Confounding factors included age, BMI, and TC. The results demonstrate that the time spent participating in Buddhist activity was negatively associated with both SBP (β = −0.112, P < 0.01) and DBP (β = −0.120, P < 0.01), which suggest a beneficial effect of greater time spent participating in Buddhist activity.

**Table 7:** Logistic regression analysis of Buddhist activity and hypertension.

	Univariate model			Multivariate model 1			Multivariate model 2		
	OR	95% CI	P value	OR	95% CI	P value	OR	95% CI	P value
Age (year)	1.055	1.044–1.067	0.000	1.087	1.068–1.107	0.000	1.077	1.048–1.107	0.000
Gender (male vs female)	0.869	0.625–1.208	0.403	0.941	0.563–1.573	0.817	0.492	0.230–1.055	0.068
BMI (kg/m^2^)	1.036	0.998–1.076	0.067	1.131	1.071–1.195	0.000	1.050	0.964–1.144	0.259
Family history of hypertension	2.478	1.210–5.077	0.013	2.962	1.055–8.318	0.039	4.407	1.206–16.104	0.025
Diabetes mellitus	1.040	0.224–4.833	0.961	—	—	—	1.086	0.131–8.973	0.939
TC	1.639	1.365–1.968	0.000	—	—	—	1.240	0.921–1.671	0.157
Buddhist activity time < 8 h/d	—	—	—	—	—	—	—	—	—
Buddhist activity time 8–10 h/d	0.341	0.201–0.579	0.000	0.890	0.426–1.860	0.758	0.779	0.321–1.889	0.580
Buddhist activity time 10–11 h/d	0.199	0.113–0.349	0.000	0.436	0.202–0.941	0.034	0.287	0.105–0.785	0.015
Buddhist activity time ≥ 11 h/d	0.116	0.063–0.213	0.000	0.238	0.109–0.522	0.000	0.078	0.023–0.268	0.000

Model 1 includes confounding factors such as age, gender, BMI, and family history of hypertension.

Model 2 includes confounding factors such as age, gender, BMI, family history of hypertension, TC, and diabetes mellitus.

Interestingly, When vegetarian and Buddhist activity time were put into the same logistic regression equation together, longer Buddhist activity time was still significantly protective (Q2 vs Q1, OR 0.766, CI 0.314–1.865, P = 0.557; Q3 vs Q1, OR 0.284, CI 0.104–0.780, P < 0.05; Q4 vs Q1, OR 0.073, CI 0.021–0.255, P < 0.01) while the role of vegetarian turned to be non-significant (OR 0.749, CI 0.364–1.541, P = 0.432) when age, gender, BMI, family hypertension history, DM and cholesterol were controlled.

## Discussion

In our study, the comparison between Tibetan Buddhists and ordinary residents demonstrates the protective role of Buddhism against hypertension in a Tibetan population. Moreover, as a Buddhism-related factor, vegetarian diet highly approximates to be protective against hypertension. Another important Buddhist behaviour is Buddhist activity participation, and longer participation time was found to be associated with a decreased prevalence of hypertension and lower BP. Furthermore, Buddhist activity was further suggested to be more vital than vegetarian diet in Tibetan hypertensive protection. Although it does not present a fully developed method for hypertensive prevention, this study suggests that religious behaviours including practising a vegetarian diet and engaging in Buddhist activity may be popularized to the general population as an alternative strategy to medicine. To our knowledge, this is the first time that an effect of Buddhist activity on hypertension has been revealed.

Studies about religion and hypertension use heterogenous methods to measure religion^5,14^. Specifically, the dependent variables can be divided into two categories. One category assesses specific aspects of religion such as attendance frequency^8–10^ and subjective religiosity^11–13^, while the second category systematically treats religion as an integrated whole. Our investigation adopted the latter method, which can demonstrate the effects of religion more comprehensively.

The protective effects of religion against hypertension revealed by our research have been supported by other studies that also compared hypertension between full-time religious staff (monks, nuns, etc.) and ordinary residents. In a 32-year prospective study conducted by Timio *et al*. comparing changes in BP between 144 nuns in a secluded order in Italy and 138 healthy lay women living nearby, BP remained virtually unchanged among the nuns while the women of control group showed increase in BP over time^17^. There was a significant difference of more than 30/15 mmHg between the two groups during the follow-up period, although the baseline data, BMI increase and menopause ages of the two groups were comparable^17^. Another study by Kunin *et al*. in the United States also found significantly lower BP in Roman Catholic nuns than in working women, with an impressive magnitude of 4 to 7 mmHg difference^18^. A third study was carried out among 984 monks and 1042 Tibetan residents in the Gannan Tibetan autonomous district of China, in which the overall prevalences of hypertension and BP level in monks were significantly lower than those in local residents^15^. Meanwhile, the role of religious behaviours as mediators of these effects was partially agreed on by these investigations^15,18^. Although the study with Italian nuns did not analyse any specific behaviours of the nuns^17^, the other two investigations about American nuns^18^ and Chinese monks^15^ analysed not taking contraceptive pills and consuming a healthy diet, respectively, as contributing behaviours^15,18^. Furthermore, the study with Chinese monks suggested a protective effect of a healthy diet against hypertension^15^. However, both studies only focused on lifestyle-related behaviours and did not pay sufficient attention to behaviours highly associated with the spiritual meaning of religion^15,18^. In addition, Li *et al*. only demonstrated differences between two populations in terms of hypertensive prevalence and diet components, without analysing the effect of diet on hypertension^15^.

Alongside the aforementioned studies, in another investigation conducted by Houser *et al*. among 670 church personnel in North-Eastern Congo, pastors did not demonstrate a significantly lower prevalence of hypertension than church administrators^19^. We speculate that the behaviours of priests can explain the result. In contrast to monks/nuns who have unique behaviours protecting against hypertension, the routine of priests involves many stressful secular affairs such as listening to peoples’ sadness and confessions, providing people forgiveness and encouragement^20,21^, teaching people the faith’s wisdom^22^, and performing sacred rituals at marriages or funerals^21^. Since priests are also confronted with the pressures of a social life, their risk of hypertension is comparable to that of ordinary people. The findings of that study indirectly support the role of specific behaviours as the mediator between religion and hypertension.

In our study, the protective effect of the vegetarian diet on hypertension was very close to be statistically significant in multiple logistic regression analysis. Therefore, we still consider vegetarian diet to be an independently protective factor against hypertension since its effect should be clearly proven with an adequate sample size. This agrees with the findings of previous studies, which indicated lower BP^16,23,24^ and lower prevalence of hypertension^16^ in vegetarians. The benefits of vegetarianism are generally attributed to decreased BMI^23,24^. For example, the linear correlation between vegetarian and BP disappeared after adjustment for BMI in the Indian Migration Study(IMS) study^23^, and the protective role of vegetarian was also mostly owing to lower BMI in the EPIC-Oxford cohort study^24^. However, unlike previous studies, this survey demonstrated a preventive role of vegetarian diet against hypertension independent of BMI, lipid level and blood glucose level. The underlying mechanism is not clear. On the one hand, some hormones such as adiponectin or leptin may exert effects on hypertension independent of BMI^25,26^. On the other hand, during the long period of adaptation to the harsh environment of the plateau, specific changes may emerge in genes or pathways adjusting BP or glucose/lipids metabolism, which need further investigation.

In contrast to the findings of our study, a study conducted among 706 females from health check-up clinics at a Buddhist hospital in Taiwan found no association between Buddhist vegetarianism and reduced hypertension/systolic BP^27^. This may be attributed to the small difference in food consumption between vegetarians and non-vegetarians since all the subjects were females, who eat less than males.

Moreover, Buddhist activity which was unique in Tibetan monks/nuns was found to be significantly and independently protective for Tibetan hypertension, and its role was suggested to be more important than vegetarian diet in this study. In fact, Buddhist activity belongs within the scope of meditation, which refers to a family of practices sharing many common points^28^. Five chief components are used to define a practice as meditation^29^. The components are as follows: the practice (1) utilizes a specific and clearly defined technique, (2) involves muscle relaxation somewhere during the process, (3) involves logic relaxation (i.e., not ‘to intend’ to analyse the possible psychophysical effects, not ‘to intend’ to judge the possible results, not ‘to intend’ to create any type of expectation regarding the process), (4) is a self-induced state, and (5) uses a self-focusing skill or ‘anchor’ for attention^29^. Buddhist activity has these five components. In detail, during the process, the lotus pose with a relaxed body, closed eyes and regular respiration should be maintained, and Buddhists should also be focused on imagining the Buddha and reciting Buddhist scriptures. Specifically, Buddha represents the essence of the world, and the content of Buddhist scriptures include praying for Buddhist wisdom, blessing living creatures, and confessing crimes and faults. Practitioners enter a state of self-induced relaxation through sitting quietly and repeating mantras because they are consistently concentrated on profound thinking instead of speculating about any particular problems.

Meditation itself, without being incorporated into any explicit religious framework, has been found to significantly reduce BP and hypertensive risk^30^, partially due to stress relief^28,30,31^. As a science of the mind, Buddhism can bring psychological relaxation through kindness, forgiveness, a sense of belonging, a feeling of security, a removal from obsession, etc. Therefore, the beneficial effect of Buddhist meditation on hypertension is explainable since it combines the effects of meditation and the Buddhist spirit. Our result is supported by the results of an interventional study including 52 Thai males (20–25 years old) who practised Buddhist meditation for 2–4 hours per day and 30 Thai males (the same age) who did not practise meditation, in which the meditation group exhibited greater decreases in systolic and diastolic blood pressure at the end of a six-week period^32^. While that study demonstrated the short-term effects of Buddhist meditation on BP change, our research further reveals the long-term effect of Buddhist meditation on hypertension. This has great potential for hypertension prevention if this practice can be spread to the general population. More relevant evidence is required in the future.

In addition, although Buddhists had lower rate of hypertensive awareness and treatment than ordinary residents, the blood pressure control rate in treated hypertensive Buddhists was significantly higher than that of residents. Further analysis demonstrated that Buddhists were more likely to have BP controlled compared with residents. Therefore, it’s suggested that religion benefits not only hypertensive prevention but also BP control in hypertension patients under antihypertensive treatment. Religion’s role in lowering elevated BP is agreed by previous investigation which also found that religious beliefs served to enhance ability to cope with having hypertension^33^. There are two explanations. First of all, in fact, hypertensive prevention and BP control are two manifestations of the blood pressure lowering effect of religion. Specifically, blood pressure is a continuum, thus reduction of BP is represented as prevention of hypertension in normotensive subjects and assistance of reaching target BP in treated hypertension patients respectively. In the aspect of lifestyle mediators of religion on hypertension, effect of vegetarian diet is demonstrated as both hypertensive prevention in general population^16^ and BP reduction in hypertensive subjects^34^. Buddhist meditation also can cause BP decrease both in normotensive^35,36^ or hypertensive participants^37,38^, which can respectively contribute to hypertensive prevention and BP control. Secondly, religion is also suggested to improve antihypertensive medication adherence by previous researches^39,40^. Due to the insufficient sample size of participants under antihypertensive treatment and the deficiency in data of medication adherence, analysis about Tibetan Buddhism on BP control as well as its mediators should be further conducted in future investigation.

Our research had some limitations. First, since the Buddhists and residents are unfamiliar with modern medicine, preventing some of them from participating the survey, there may have been a selective bias that could result in an imprecise comparison between the two groups. The sample size was adequate according to the calculation, and the non-participation rates of both groups were similar. Second, this was a cross-sectional study; thus, further cohort or interventional studies are needed to define the causality. Finally, although psychological relaxation should be a key mediator in the association between Buddhist activities and blood pressure, the psychological status of the subjects was not examined in this study. Investigation about it is requisite in the future.

## Methods

This was a cross-sectional study.

### Research area

The Ganzi Tibetan Autonomous Prefecture of Sichuan Province is adjacent to the southeast border of Tibet. It is the second largest Tibetan area in China, with an average altitude of 3500 m. The total population is 1.11 million, and 80% of the residents are Tibetans.

This study was conducted in two world-famous Tibetan Buddhist colleges and several surrounding villages and towns. One college was the Seda Larong Buddhist Institute in Seda County, which is the largest Tibetan Buddhist college in the world, with almost 40000 monks and nuns. The other college was the Yaqing Temple in Baiyu County, with almost 20000 Buddhists. Several villages and towns around the two locations were surveyed.

### Subjects

The enrolment period was from September 2016 to October 2017. The monks and nuns were recruited by cluster random sampling using the residential zone of the Buddhist colleges as the sampling unit. Tibetan residents were enrolled from several randomly chosen villages and towns nearby. The Buddhists were informed about the study by the principal of each chosen zone, and all the residents were informed by door to door visits. The address and ID number of each subject were recorded.

The included subjects were adult Tibetan Buddhists who had lived in one of the Buddhist colleges for ≥6 months or local Tibetan residents who had lived in the villages and towns near these Buddhist colleges for ≥6 months.

This study was approved by the ethics committees of the West China Hospital. Informed consent was obtained from each participant before recruitment. This study was performed according to the principles of the Helsinki Declaration for medical research involving human subjects.

### Data collection

#### Questionnaire

Demographic data (e.g., age, gender), clinical data (e.g., history of diabetes mellitus and hypertension, family history of hypertension, use of antihypertensive drugs), and lifestyle information (e.g., smoking status, alcohol consumption, exercise and diet) were obtained via a self-administered questionnaire. Since Tibetan residents have their own language that is different from Mandarin, each investigator was assigned a translator. The investigators and translators were medical workers from the West China Hospital and the local health system. Before the survey, all the staff members were uniformly trained.

#### Physical examination

The physical examination involved assessments of height, weight, BP, and pulse rate. In a room kept at 25 °C, after resting in a sitting position for 10 minutes, BP and heart rate (HR) were measured in the right arm by a trained doctor using calibrated electronic BP monitor (HEM-770A, Omron Healthcare Co. Ltd, Kyoto, Japan). The measurement of BP was conducted in a standard way^41^. Two consecutive BP readings were obtained with a 1-minute interval between them. The average of the two readings was used for analysis. However, if the difference between the two BPs was more than 10/5 mmHg, another measurement was taken, and the average was calculated using the last two records.

#### Biochemistry tests

In the morning, after 12 hours of fasting, blood samples were drawn from the antecubital vein into non-anticoagulant tubes. After centrifugation at 3000 r/min, the serum was removed. All the samples were stored at <4 °C until they were transported to the West China Hospital, where they were stored at −80 °C. The biochemical indicators including serum TC, low density lipoprotein cholesterol (LDL-C), high density lipoprotein cholesterol (HDL-C), and triglycerides (TG) were measured at the laboratory of the West China Hospital with an automatic biochemical analyser (HITACHI 7600, MD, Japan). Considering the degradation of fasting plasma glucose over time, we measured fingertip blood glucose (ACCU-CHEK® Performa Nano, Roche).

### Related definitions

#### Regular definitions

Hypertension was defined as having a SBP of at least 140 mmHg and/or a DBP of at least 90 mmHg and/or currently taking antihypertensive medications^41^. Diabetes mellitus was defined as one of the following: (1) fasting fingertip glucose ≥7.0 mmol/L, (2) random fingertip glucose ≥11.0 mmol/L, (3) a positive response to the question, ‘Has a doctor ever told you that you have diabetes?’, or (4) the current use of insulin or oral hypoglycaemic agents. ‘Smoking’ was defined as currently consuming an average of 1 cigarette per day. ‘Alcohol intake’ was defined as the current average consumption of at least 50 g alcohol per day. ‘Physically active’ was defined as exercising 3 or more times per week, with at least 30 minutes of exercise each time. BMI was calculated by dividing each patient’s weight (kg) by the square of his or her height (m^2^). BMI < 25 kg/m^2^ was defined as normal, BMI ≥ 25 kg/m^2^ was defined as overweight, and BMI ≥ 30 kg/m^2^ was defined as obese. A family history of hypertension was recorded if any one of the subjects’ parents or siblings had hypertension.

#### Vegetarian

The definition used in this study is the same as that in Petersen *et al*.^16^. Specifically, vegetarians were defined as those who have specific restrictions on the frequency of their intake of meat, fish and dairy. They can be divided into vegans, lacto-ovo vegetarians and partial vegetarians. Vegans are those who eat meat, fish and dairy less than once per month. Lacto-ovo vegetarians eat meat and/or fish less than once per month and eat dairy more than once per month. Partial vegetarians include pesco-vegetarians who eat meat less than once per month and fish at least once per month and semi-vegetarians who eat meat at least once per month but who eat fish and meat less than once per week.

#### Buddhist activity

The Buddhist activity was carried out in a seated position. Physically, it should be done in a standard posture similar to lotus pose with crossed legs, closed eyes, and regular respiration. While in this position, the person should imagine the image of Buddha, comprehend the essence of the world, recite scriptures, pray for living creatures, confess their faults, etc. The whole time spent on Buddhist activity per day was acquired from the questionnaire.

### Statistical analysis

According to previous studies, the prevalence of hypertension in Tibetan Buddhists is 19.3%^15^, while the prevalence in Tibetan residents in Sichuan Province is 45.7%^42^. The minimum sample size needed was calculated to be 79 in both groups, with a power of 0.9 and a 2-tailed α-value of 0.05.

Continuous data are presented as the means ± standard deviations (SDs) if they demonstrated normal distributions and as medians and quartiles if they were not normally distributed. Categorical variables are described as frequencies and percentages. For continuous variables with normal distributions, Student’s t test was used to distinguish differences between the two groups, and analysis of variance (ANOVA) was applied to determine differences among ≥3 groups. The least significant difference (LSD) test for pairwise comparisons was used when needed. Continuous data with non-normal distributions were analysed by nonparametric tests. The Chi-square test was used for categorical data. The age-standardized hypertensive prevalence rate was calculated according to the national age weight of the Chinese population, which was published by the Ministry of Health in 2000. To determine the factors associated with hypertension, multiple logistic regression analysis was performed. Analysis of covariance was used to compare vegetarians and non-vegetarians, using age as a covariate. Multiple linear regression models were used to identify the relationship between Buddhist activity time and BP. A 2-sided significance level of 0.05 was used. Statistical analyses were performed using SPSS software (version 19.0, SPSS Inc, Chicago, IL, USA).

### Data availability

All raw experimental data used in this study are available from the corresponding author upon request.

## Conclusion

A protective role of religion against hypertension is suggested since Tibetan Buddhists had a significantly lower risk of hypertension than ordinary residents. The religion-related behaviours including vegetarian diet and Buddhist activity were mediators of this protective effect that can be applied to the general population. Furthermore, Buddhist activity is suggested to be more important than vegetarian diet in the protection of Tibetan hypertension. These preliminary findings require confirmation in larger clinical trials.
